# Editorial Perspective: Advancements in Microfluidics and Biochip Technologies

**DOI:** 10.3390/mi16010077

**Published:** 2025-01-11

**Authors:** Hyunil Ryu, Tae-Joon Jeon, Sun Min Kim

**Affiliations:** 1 Biohybrid Systems Research Center (BSRC), Inha University, 100 Inha-ro, Michuhol-gu, Incheon 22212, Republic of Korea; hyunil.ryu@gmail.com; 2 Department of Biological Engineering, Inha University, 100 Inha-ro, Michuhol-gu, Incheon 22212, Republic of Korea; 3 Department of Biological Sciences and Bioengineering, Inha University, 100 Inha-ro, Michuhol-gu, Incheon 22212, Republic of Korea; 4 Department of Mechanical Engineering, Inha University, 100 Inha-ro, Michuhol-gu, Incheon 22212, Republic of Korea

Microfluidics and biochip technologies continue to play a key role in driving innovation across biomedical, environmental and engineering disciplines. Recent advances have contributed significantly to addressing challenges in diagnostics, therapeutics and environmental monitoring. This review summarizes key findings from recent publications, highlighting developments in four main areas: (1) material and fabrication innovations [1,2,3,4], (2) droplet-based platforms [5,6,7,8,9,10,11], (3) sensitive and portable biosensors [12,13,14,15,16,17,18], and (4) personalized cell chips [19,20,21,22,23] and artificial intelligence (AI) integrated with biochips [24,25,26,27,28].

1. Material and manufacturing innovations: Advances in materials and manufacturing processes have driven the development of next-generation microfluidic platforms. Rodrigues et al. highlighted the potential of cyclic olefin copolymers (COCs) as alternatives to polydimethylsiloxane (PDMS) [2]. COCs exhibit low autofluorescence and high thermal stability, enabling rapid prototyping and scalable production via hot embossing. These platforms have demonstrated efficacy in applications such as DNA hybridization and high-throughput assays. Lee et al. presented a method for fabricating robust glass-based biochips using micro-carbon molds created by electrical discharge machining (EDM) [3]. This approach improves optical and mechanical properties and provides scalability for glass microstructures. These advances highlight the potential of novel materials and techniques to overcome limitations associated with traditional materials, thereby broadening the scope of microfluidic applications.

2. Droplet-based platforms: Droplet-based microfluidic systems represent a paradigm shift from traditional channel-based approaches. Tanev et al. developed the modular BiowareCF platform, which is capable of programmable and reusable operations [10]. By integrating fluid components with digital programmability, the system bridges experimental workflows and computational models, enabling scalable and adaptable designs. Zhang et al. presented dropletronic devices using surfactant-based hydrogel droplets to mimic electronic devices such as diodes and transistors [11]. These devices also exhibit neuromimetic properties, enabling real-time interactions with human cardiomyocytes for electrophysiological signal recording. These innovations highlight the transformative potential of droplet-based platforms in biosensing and biointerface applications.

3. Sensitive and portable biosensors: The development of biosensors with enhanced sensitivity and portability remains a focus. Zhu et al. combined localized surface plasmon resonance (LSPR) and quartz crystal microbalance (QCM) technologies to create a hybrid sensor with exceptional sensitivity for the detection of RNA viruses [16]. This device has demonstrated its utility in public health emergencies such as the COVID-19 pandemic. Wang et al. used loop-mediated isothermal amplification (LAMP) on a microfluidic biochip to achieve rapid and accurate detection of *E. coli* in environmental samples [17]. Li et al. developed a graphene oxide-based biochip for the detection of harmful algal blooms (HABs) at concentrations above 10^8^ aM [18]. This portable platform combines high sensitivity with rapid response times. These systems emphasize the role of microfluidics in real-time diagnostics and field applications ranging from public health to environmental monitoring.

4. Personalized cell chips: Microfluidics has increasingly become a cornerstone of personalized medicine due to its small, cost-effective devices. Prabowo et al. developed a microfluidic chip for the detection of stroke biomarkers using magnetic nanobeads to label cell-specific fibronectin (c-Fn) and MMP9 [21]. This system has shown high sensitivity and specificity, highlighting its potential for emergency stroke diagnosis. In addition, label-free research is also actively underway. Sitkov et al. have advanced label-free diagnostics by integrating peptide aptamers for protein binding, enabling real-time, cost-effective detection with high clinical applicability [22]. Stollmann et al. presented an optofluidic platform that combines digital holography and supported lipid bilayers to perform label-free molecular fingerprinting of extracellular vesicles (EVs) [23]. This technology advances biomarker discovery and EV profiling, demonstrating its utility in precision medicine.

5. Artificial intelligence (AI) integrated with biochips: Recent innovations in deformability-based biochips, neuromorphic-enabled systems, and deep learning-enhanced platforms exemplify the transformative potential of this convergence. Hua et al. integrated a deep learning framework named ATMQcD [26]. This biochip uses cellular deformability as a biomarker to classify cancer metastasis subtypes with 92.4% accuracy. It utilizes constriction-based cytometry and power-law rheology modeling to assess cell stiffness, achieving an 89.5% accuracy rate in distinguishing between cancer cells and leukocytes. Another notable advancement is the Neuromorphic-Enabled Video-Activated Cell Sorting (NEVACS) system, developed by He et al., which integrates microfluidics, event-based cameras, and spiking neural networks (SNNs) [27]. NEVACS captures 3D information about cells’ positions and movements, allowing it to sort 1000 cells per second with almost no errors. Tong et al. took biochip technology using AI even further by creating a portable microwell array that can learn deep learning algorithms to detect and measure different types of *Cryptococcus* [28]. This platform uses the CRISPR–Cas12a system and a “segment anything model” (SAM) algorithm for detecting pathogens. The platform’s exceptional specificity (100%) and ability to detect minuscule quantities (0.5 pM) of pathogens highlight the significant advancements made by integrating computer intelligence with microfluidic platforms.

The convergence of materials science, systems integration and application-driven research underscores the innovative potential of microfluidic and biochip technologies. Despite significant progress, challenges remain in standardization, cost reduction and multi-scale integration. Addressing these priorities will improve the versatility and manufacturability of devices, enabling high-impact innovations in healthcare, environmental sustainability and industrial efficiency.
